# Core outcome sets and systematic reviews

**DOI:** 10.1186/s13643-016-0188-6

**Published:** 2016-01-20

**Authors:** Mike Clarke, Paula R. Williamson

**Affiliations:** Northern Ireland Network for Trials Methodology Research, Centre for Public Health, Institute of Clinical Sciences, Block B, Queen’s University Belfast, Royal Hospitals, Grosvenor Road, Belfast, BT12 6BJ UK; North West Hub for Trials Methodology Research, University of Liverpool, 1st floor Duncan Building, Daulby Street, Liverpool, L69 3GA UK

## Abstract

Systematic reviews seek to bring together research evidence to answer the question for the review. The reviewers usually wish to compare, contrast and, if appropriate, combine the findings of the existing research studies. However, these intentions are often thwarted by inconsistencies in the outcomes that were measured and reported in the individual studies. This, in turn, makes it difficult for readers of the review to use it to make informed decisions and choices about health and social care. One solution is for trials in a particular topic area to measure and report a standardised set of outcomes, which would then be used in the review. Core outcome sets are a means of doing this, providing an agreed standardised collection of outcomes for measuring and reporting for a specific area of health. In this commentary, we argue for greater involvement of systematic reviewers in the development and implementation of core outcome sets. This might help with, for example, the selection of outcomes to include in the Summary of findings tables that provide users of the review with the key quantitative findings. Consideration of core outcome sets when reviewers register their topics with Cochrane Review Groups or in PROSPERO would also help reviewers to plan their reviews. A greater uptake of core outcome sets across research, including systematic reviews, would help towards the ultimate aim of improving health and well-being through improving health and social care.

## Background

Systematic reviews seek to bring together the evidence from research that already exists, to answer the question for the review. In many cases, the aim is to combine the findings of the research studies in a meta-analysis, thereby increasing statistical power and precision of the effect estimate and supporting the conduct of additional analysis, such as the investigation of the effects in different subgroups. Even if there is no desire to combine the results of the studies, reviewers might wish to compare and contrast the included studies, to explore the effects of clinical and methodological heterogeneity. However, these intentions are often thwarted by inconsistencies in the outcomes that were measured and reported in the individual studies. It might not be possible to compare, contrast or combine the results of the individual studies because those results are presented for different outcomes. This contributes to waste in research [1]. It means that many reviews which would otherwise be able to pool results and generate robust effect estimates are unable to do much more than summarise the findings of each of the eligible studies as a separate piece of information. This, in turn, makes it difficult for readers to use the review to make informed decisions and choices about health and social care.

One solution, which would also help with streamlining the systematic reviewing process [2], is for all trials in a particular topic area to measure and report a standardised set of outcomes and for these outcomes to be used in the review. This would not limit the ability of researchers to examine other outcomes that might be of particular interest for their research but would mean that every trial could contribute useful data to the analyses of the key outcomes. One means to achieve this is through the development of core outcome sets, and we present the case for greater involvement of the systematic review community in their development and implementation in this commentary. It builds on an earlier commentary, encouraging greater use of core outcome sets in trial registries [3].

## Main text

### Core outcome sets

Core outcome sets are an agreed standardised collection of outcomes that should be measured and reported for a specific area of health. These sets represent the minimum that should be measured and reported in all clinical trials of a specific condition and are also suitable for use in other types of research and clinical audit [4]. Although there are examples of core outcome sets going back more than 30 years [5], and some well-established ones such as Outcome Measures in Rheumatology (OMERACT) for rheumatoid arthritis [6], they are still relatively rare. A systematic review, with searches up to 2013, found just under 200 in the published literature, across a wide range of areas but with many notable gaps, including for common conditions such as diabetes [7]. In some areas of health and social care, researchers might have already reached a stage where all (or almost all) studies measure more or less the same set of outcomes, but a formal process of agreeing on a core outcome set might still help to cement this informal consensus. This could also reinforce the important point that the standardisation which is achieved with a core outcome set is not intended to stifle innovation [4]. The outcomes in the set should represent the minimum to be collected in all trials, and researchers should continue to measure and report additional outcomes of particular relevance to their topic. Likewise, systematic reviewers should continue to seek data on those outcomes of particular relevance to their review, above and beyond those in the core outcome set.

If core outcome sets are to contribute to improvements in health and social care, by helping patients and the public, practitioners and policy makers to make better decisions about interventions, they need to contain the outcomes that really matter to these stakeholders.

They need to be developed in a collaborative way, with appropriate representation from key stakeholders, including practitioners, patients and others users of health and social care services; as well as researchers, policy makers and those who fund services and research [3]. Systematic reviewers from the relevant area of health or social care should be among these stakeholders in the development of core outcome sets. They may be able to help in relation to the review of outcomes previously measured in studies in the particular area and bring to the consensus process the perspective of the researchers who might have struggled most with inconsistencies in outcomes in the existing literature. Their involvement may facilitate the incorporation of these views into the process and help to identify ways to overcome some of the problems encountered by reviewers when selecting outcomes for their reviews and by users when reading reviews. Furthermore, explicit engagement of systematic reviewers in the development of core outcome sets may encourage subsequent uptake of these sets by this group of researchers.

In order to facilitate the positive impact of core outcomes sets on health and social care, the COMET (Core Outcome Measures for Effectiveness Trials) Initiative (www.comet-initiative.org) is seeking to support others in the development and uptake of core outcome sets across all areas of health and social care [8]. Since its launch in 2010, COMET has compiled resources to help people developing core outcome sets and a freely accessible database of published core outcome studies, drawing on the aforementioned systematic review [7] and subsequent updating using the same search strategy [9]. Furthermore, recognising the challenges that people reading reports of a core outcome set face when trying to decide whether to adopt an existing core outcome set or develop a new one, work is underway to develop a reporting guideline, using an international consensus process [10]. It is also important to note that the development of a core outcome set which focuses on the *what* to measure may need to be followed by decisions about *how* and *when* to measure. Heterogeneity in how outcomes have been measured or the timing of those measurements can undermine efforts to facilitate the work of systematic reviewers who are trying to compare, contrast and combine the results of multiple studies even if the outcomes themselves are consistent across the studies. This is an area of ongoing collaboration between COMET and COSMIN (COnsensus-based Standards for the selection of health Measurement INstruments) [11].

### Core outcome sets in systematic reviews

Currently, the explicit use of core outcome sets in systematic reviews seems rare. For example, a survey of all Cochrane Reviews that were first published in full in 2007 (387 reviews), 2011 (401), and 2013 (439) did not identify any that cited a core outcome set as influencing the choice of outcomes to investigate in the review but found that these 1227 reviews included a total of nearly 9800 outcomes [12, 13]. Considering the new reviews from 2013 only [13], the number of specified outcomes in each review ranged from just one in three reviews to 62 in a review which examined different types of dietary advice for women with gestational diabetes and specified short- and long-term outcomes for both mother and child [14]. The median number of outcomes was 7. The 439 reviews specified a total of 3644 outcomes in their methods sections. After excluding the 65 reviews that did not have any included studies, 68 % (2134/3142) of these specified outcomes were reported in the “Results” section of the review. Of the 1008 non-reported outcomes across all reviews that had studies in them, 77 % (775/1008) had not been reported because they were not measured in the studies included in the review or insufficient data were available. No clear reason was found in the text of the review for the non-reporting of the remaining 23 % (233/1008) [13].

One of the ways in which core outcome sets might be particularly useful for Cochrane Reviews [15] and other systematic reviews is in the selection of outcomes to include in the Summary of findings tables developed by the Grading of Recommendations Assessment, Development and Evaluation (GRADE) working group to summarise the results for important outcomes and the quality of this evidence [16]. For Cochrane Reviews, it is recommended that these tables include seven or fewer outcomes, and they have been shown to improve readers’ understanding and speed of retrieval of the findings of the review [17]. They were introduced to Cochrane Reviews in 2008 and were included in 112 (31 %) of the 361 full reviews published for the first time in 2011 and containing at least one included study, rising to 57 % (216 of 375 new reviews) in 2013 [13].

### Using core outcome sets when registering a systematic review

An important opportunity for systematic reviewers to make use of core outcome sets, and to be clear about doing so, is when choosing the outcomes to assess in their review. They can make this explicit in the protocol for their review and in its prospective registration. Since its inception, Cochrane has required authors of Cochrane Reviews to have their proposed review approved by the relevant Cochrane Review Group and then prepare and publish the protocol for their review. Since 2011, it has been possible for all systematic reviewers to register their review at their outset in PROSPERO, a free-to-use registry for systematic reviews developed and run by the Centre for Reviews and Dissemination in the University of York, UK [18]. PROSPERO recorded its 10,000th entry in 2015, and as in the protocols for Cochrane Reviews, reviewers are able to list the outcomes that they will attempt to assess in the included studies in their registry entry. They could mention their intended use of a core outcome set, as we have suggested for trial registries such as International Standard Randomised Controlled Trial Number (ISRCTN) [3]. It would also allow the reviewers to specify any additional outcomes that they are interested in. Specifying the outcomes from the core outcome set, and using the terms for these outcomes that were used in that core outcome set, would facilitate searching of PROSPERO and the protocols for Cochrane Reviews. Another benefit from considering the core outcome set in the design and registration of their review is that this might help reviewers to choose the outcomes to include in a Summary of findings table. This might also help to focus their subsequent actions, including the seeking of data that are not available in the reports of eligible studies so that they obtain information that is as complete as possible for these outcomes. When others inspect the registry entry for a review, the explicit use of a core outcome set will help them to decide if the eventual review is affected by selective reporting bias because the review authors have not included all the outcomes from the core outcome set when presenting their results [19].

## Conclusion

Systematic reviewers are often the researchers who struggle most because of inconsistencies in the outcomes that have been measured and reported in trials of health and social care. This hampers their ability to resolve the uncertainties that they seek to address in their reviews. They can help to overcome these problems by becoming involved in the development of core outcome sets and then using them as a foundation for the outcomes to include in their review and summaries, such as the Summary of findings tables [14]. We welcome comments on these suggestions and those that we made earlier for trial registries [3], with the overall intention of helping reviewers and other researchers achieve their ultimate aim of improving health and well-being through improving health and social care.

## Abbreviations

COMET: Core Outcome Measures in Effectiveness Trials
GRADE: Grading of Recommendations Assessment, Development and Evaluation
ISRCTN: International Standard Randomised Controlled Trial Number
OMERACT: Outcome Measures in Rheumatology
